# Transactions—National Dental Association

**Published:** 1914-06-15

**Authors:** 


TRANSACTIONS—NATIONAL DENTAL ASSOCIATION. The transactions of the Kansas City Meeting and the Southern Branch meeting at Old Point Comfort, is just published by the "Cosmos Press." It is in the usual form, and as its contents has already appeared in the pages of the Dental Cosmos it requires no reviewing. If the proceedings for coming meetings are printed in the Associations Journal, this will probably be the last issue in this form of the proceedings of this society.

